# Corrigendum

**DOI:** 10.1002/ccr3.6731

**Published:** 2022-12-13

In the article entitled “Benign or premalignant? Idiopathic granulomatous mastitis later diagnosed as ductal carcinoma breast cancer: Case report and review of literature” that has been published in Volume 10 Issue 9 of Clinical Case Reports, a paragraph had been omitted inadvertently in the published version.

The following paragraph is added as the last paragraph in “Case Presentation” section:

The physical examination revealed erythema and edema in the inner lower quarters of the left breast. There was a firm lobulate large mass (approximately 10 cm) in this part of the breast with three palpable lymph nodes in the left axilla. The ultrasonography revealed a micro‐lobulated large solid cystic mass (6.5 × 9.5 cm) at 5:00 to 9:00 o'clock in the sub‐areolar region. The solid part of the mass showed moderate peripheral and internal vascularity (Figure 3). The three enlarged lymph nodes of the left axillary measured approximately 3 cm (BIRADS5). The mammography indicated partially obscured increased density mass in the left breast's lower inner, measuring about 10 × 9.5 cm (Figure 4). There were several enlarged lymph nodes in the left axilla, and the largest one was about 4 cm. The histopathological assessment revealed a grade III invasive ductal carcinoma (Figure 5). Patient received neoadjuvant chemotherapy with four cycles AC (doxorubicin 60 mg/m^2^ and cyclophosphamide 600 mg/m^2^) followed by four cycles of paclitaxel (175 mg/m^2^) that resulted in decrease in tumor size. After eight cycles of chemotherapy, the patient underwent a simple mastectomy and sentinel lymph node biopsy (SLNB). The patient was discharged 24 h after the operation. According to the pathology report, there was residual invasive ductal carcinoma with the largest focus of tumoral nest about 6 mm in the fibrotic bed. The result of SLN was negative for malignancy. However, two lymph nodes from the tail of the breast were involved in invasive ductal carcinoma. Therefore, patient received radiotherapy with a dose of 5000 cGy in 25 fractions to the breast, axilla, and supraclavicular nodes.

The publisher apologizes for the error.
